# Acatalasemic mice are mildly susceptible to adriamycin nephropathy and exhibit increased albuminuria and glomerulosclerosis

**DOI:** 10.1186/1471-2369-13-14

**Published:** 2012-03-25

**Authors:** Keiichi Takiue, Hitoshi Sugiyama, Tatsuyuki Inoue, Hiroshi Morinaga, Yoko Kikumoto, Masashi Kitagawa, Shinji Kitamura, Yohei Maeshima, Da-Hong Wang, Noriyoshi Masuoka, Keiki Ogino, Hirofumi Makino

**Affiliations:** 1 Department of Medicine and Clinical Science, Okayama University Graduate School of Medicine, Dentistry and Pharmaceutical Sciences, 2-5-1 Shikata-cho, Kita-ku, Okayama 700-8558, Japan; 2 Center for CKD and Peritoneal Dialysis, Okayama University Graduate School of Medicine, Dentistry and Pharmaceutical Sciences, 2-5-1 Shikata-cho, Kita-ku, Okayama 700-8558, Japan; 3 Center for iPS Cell Research and Application, Kyoto University, Kyoto, Japan; 4 Public Health, Okayama University Graduate School of Medicine, Dentistry and Pharmaceutical Sciences, 2-5-1 Shikata-cho, Kita-ku, Okayama 700-8558, Japan; 5 Department of Life Science, Okayama University of Science, 1-1, Ridai-cho, Kita-ku, Okayama 700-0005, Japan

## Abstract

**Background:**

Catalase is an important antioxidant enzyme that regulates the level of intracellular hydrogen peroxide and hydroxyl radicals. The effects of catalase deficiency on albuminuria and progressive glomerulosclerosis have not yet been fully elucidated. The adriamycin (ADR) nephropathy model is considered to be an experimental model of focal segmental glomerulosclerosis. A functional catalase deficiency was hypothesized to exacerbate albuminuria and the progression of glomerulosclerosis in this model.

**Methods:**

ADR was intravenously administered to both homozygous acatalasemic mutant mice (C3H/AnLCs^b^Cs^b^) and control wild-type mice (C3H/AnLCs^a^Cs^a^). The functional and morphological alterations of the kidneys, including albuminuria, renal function, podocytic, glomerular and tubulointerstitial injuries, and the activities of catalase were then compared between the two groups up to 8 weeks after disease induction. Moreover, the presence of a mutation of the toll-like receptor 4 (*tlr4*) gene, which was previously reported in the C3H/HeJ strain, was investigated in both groups.

**Results:**

The ADR-treated mice developed significant albuminuria and glomerulosclerosis, and the degree of these conditions in the ADR-treated acatalasemic mice was higher than that in the wild-type mice. ADR induced progressive renal fibrosis, renal atrophy and lipid peroxide accumulation only in the acatalasemic mice. In addition, the level of catalase activity was significantly lower in the kidneys of the acatalasemic mice than in the wild-type mice during the experimental period. The catalase activity increased after ADR injection in wild-type mice, but the acatalasemic mice did not have the ability to increase their catalase activity under oxidative stress. The C3H/AnL strain was found to be negative for the *tlr4 *gene mutation.

**Conclusions:**

These data indicate that catalase deficiency plays an important role in the progression of renal injury in the ADR nephropathy model.

## Background

The degree of oxidative stress and the severity of subsequent tissue injury may depend on an imbalance between the excessive production of reactive oxygen species and the antioxidant defense. The antioxidants include the enzymes superoxide dismutase (SOD), catalase, and glutathione peroxidase (GPX), which detoxify reactive oxygen species. Catalase (E.C.1.11.1.6) is a major enzyme that catalyzes the decomposition of hydrogen peroxide (H_2_O_2_) and plays a role in cellular antioxidant defense mechanisms [1]. The main reaction of catalase is the catalytic reaction (2H_2_O_2 _→ O_2_+ 2H_2_O), which is essential for the removal of excessive H_2_O_2 _and for regulating the H_2_O_2 _concentration [2]. Catalase limits the accumulation of H_2_O_2 _generated by various oxidases in tissue, and serves as a substrate for the Fenton reaction to produce the highly injurious hydroxyl radicals. Genetic defects of catalase were first documented by Takahara [3] in Japanese patients who exhibited a deficiency of blood catalase enzyme activity (acatalasemia) [4,5]. Subsequently, an acatalasemic mouse strain (Cs^b^) was established by Feinstein, Suter, and Jaroslow [6] from the progeny of x-ray-irradiated mice.

Focal segmental glomerulosclerosis (FSGS) is a common cause of nephrotic syndrome in both children and adults [7,8]. The clinicopathological syndrome may be classified as primary, secondary or familial. The primary defect in FSGS lies in the filtration barrier of the glomeruli. Disruption of the filtration barrier results in the loss of permselectivity, and macromolecules such as albumin are allowed to enter the urine. Chen et al. [9] reported that BALB/c mice were susceptible to renal toxicity arising from the administration of the anthracycline antibiotic, adriamycin (ADR), with selective injury to podocytes resulting in severe proteinuria and progressive renal failure [9,10]. This was described as the first experimental model of FSGS in mice. The activities of antioxidant enzymes including catalase, GPX and Mn-SOD and the glutathione concentration in renal cortex were decreased by ADR nephropathy in BALB/c mice [11]. The level of nitric oxide in kidney homogenates [12], and the urinary levels of nitrite/nitrate [10] were also increased in the ADR nephropathy model. The administration of the soluble receptor for advanced glycation endproducts (AGEs) suppressed AGE generation and reactive oxygen species in the ADR nephropathy mice [13].

In the present study, we hypothesized that a defect in the antioxidant system in the form of catalase deficiency would enhance proteinuria, glomerular sclerosis, and eventually lead to the loss of renal function. This hypothesis was tested using the ADR nephropathy model, which is a well-established model of progressive FSGS, in acatalasemic mice.

## Methods

### Experimental animal protocol

Male wild-type mice (C3H/AnLCs^a^Cs^a^) and male homozygous acatalasemic mutant mice (C3H/AnLCs^b^Cs^b^) were used at the age of 8 - 10 weeks. Animals were housed in cages and fed standard chow and water *ad libitum*. ADR (10, 15 and 20 mg/kg BW) dissolved in saline was intravenously administered to both acatalasemic mutant mice and control wild-type mice [14]. In the C3H/AnL strain, the dose of 10 mg/kg BW did not induce significant albuminuria or increase mortality (Additional file 1: Figure S1*A*). The 20 mg/kg BW dose resulted in a rapid increase in mortality in both groups of mice (Additional file 1: Figure S1*C*). The ADR-induced cardiotoxicity might have led to the high mortality resulting from the higher dose of ADR [15]. A total of 55.3% of the acatalasemic mice and 59.2% of the wild-type mice survived 8 weeks after the injection of 15 mg/kg BW ADR (Additional file 1: Figure S1*B*) with a substantial amount of albuminuria. Therefore, 15 mg/kg BW ADR was considered to be a suitable concentration to investigate the effects of a functional catalase deficiency in the ADR nephropathy model. In the control mice, the same volume of saline was injected intravenously.

Mice were divided into subgroups (n = 6-15/group). Their body weight was measured at 0, 4 and 8 weeks. The mice were sacrificed at 0, 4 and 8 weeks after ADR administration, then their kidneys and hearts were harvested, washed with saline, blotted dry on gauze, and weighed as described previously [16,17]. The whole kidney weight and heart weight were expressed as a percentage of the body weight determined at the time the mice were sacrificed. Twenty-four hour urine samples were collected in metabolic cages every 4 weeks. Immediately before death, blood samples were drawn. The serum creatinine, blood urea nitrogen (BUN), and urinary albumin excretion (UAE) levels were measured, and the creatinine clearance (Ccr) was calculated as described previously [16]. The experimental protocol was approved by the Ethics Review Committees for Animal Experimentation of Okayama University Graduate School (OKU-2009226).

### Reagents and antibodies

Chemicals and reagents of analytical grade were purchased from Sigma Co. Ltd. (St. Louis, Missouri) or Wako Pure Chemical Ind. (Osaka, Japan) unless stated otherwise. The mouse monoclonal antibody to 4-hydroxy-2-nonenal (4-HNE) was obtained from Nof Life Science (Tokyo, Japan). The N-histofine MOUSESTAIN KIT was obtained from NICHIREI BIOSCIENCES INC. (Tokyo, Japan). The catalase assay kit was obtained from Cayman (Ann Arbor, Michigan).

### Light and electron microscopic studies

The kidneys were removed, fixed in 10% buffered formalin, and embedded in paraffin. Paraffin sections (3-μm thick) were stained with periodic acid-Schiff (PAS) and Masson's trichrome stain. Each tissue section was evaluated under an Olympus light microscope (Olympus, Tokyo, Japan) with a high-resolution digital camera system (Penguin 600CL; Pixera Co., Los Gatos, CA). The area of glomeruli was measured using a Microanalyzer software program (version 1.1, Japan Poladigital Co., Tokyo, Japan) [18]. Glomerulosclerosis was quantified using the percentage increase in the relative mesangial matrix area (the PAS-positive area within the glomerulus divided by the glomerular capillary area; high magnification). All mean values were calculated from 10 glomeruli. Electron microscopy was performed for the mouse kidney specimens as described previously [16,17]. A quantitative analysis was performed to count the number of podocyte foot processes per 10 μm of glomerular basement membrane in each glomerulus by electron microscopy. The mean number of podocyte foot processes was defined as the effacement score. The area of interstitial fibrosis in the cortex was evaluated with Masson's trichrome as described previously [19], with some modifications. Under low magnification, the number of casts in five randomly selected non-overlapping fields from the cortical region was counted and averaged as the cast score.

### Immunohistochemistry

To detect lipid oxidation products, paraffin sections (3-μm thick) were stained using an N-histofine MOUSESTAIN KIT as described previously [20]. A mouse monoclonal antibody to 4-HNE was used as the primary antibody (1:100 dilution). Under low magnification, five randomly selected non-overlapping fields from the cortical region were analyzed. The 4-HNE positive areas that were stained in brown were picked up on digital images, and the percentage of the 4-HNE positive area relative to the whole area of the field was calculated (% area).

### Renal catalase activity

Kidney samples were stored in a -80°C freezer until being assayed. The catalase activity in each kidney was determined by an ELISA using a catalase assay kit [21]. All procedures were performed according to the manufacturer's instructions.

### Toll-like receptor-4 gene (*Tlr4*) mutation analysis in exon 3 in wild-type (C3H/AnLCs^a^Cs^a^) and acatalasemic mice (C3H/AnLCs^b^Cs^b^)

The detection of the *Tlr4 *mutation was performed using nested-PCR as described previously [22]. The nested PCR for detection of the missense mutation, a C to A transversion (Pro712His), which was reported in the C3H/HeJ mouse strain [23], was performed using the primers shown in Additional file 2: Table S1. The primers TLR4-ex3-F and TLR4-ex3-R were used for direct sequencing of DNA extracted from the mouse kidneys.

### Statistical analyses

The data were shown as the means ± SE. The normal distribution and homogeneity of variance were checked, and logarithmic transformations were made for the variables if needed. Multiple comparisons between groups were made by Scheffe's test or the Steel-Dwass test. A Kaplan-Meier analysis and the Log-Rank statistic were used to explore the effects of ADR in both groups of mice. The statistical analysis was performed using the Excel add-in software Statcel 2 program (OMS, Tokyo, Japan) or the JMP 9 software program (SAS Institute Inc., Cary, North Carolina). P < 0.05 denoted the presence of a statistically significant difference.

## Results

### Changes in body weight, kidney weight, and heart weight in the mouse ADR nephropathy model

The ADR nephropathy model has been extensively studied in animals of the BALB/c background. Therefore, we first tried to characterize this model in our mouse strains. The body weight, relative kidney weight and relative heart weight were similar between the groups at the start of the experiment (Table 1). Both groups of mice lost a similar amount of body weight by 8 weeks after ADR administration. However, the relative kidney weight in acatalasemic mice was lower at 8 weeks than that in wild-type mice after ADR administration. The relative heart weights were similar for both groups during the experimental period.

**Table 1:** The metabolic and laboratory data of the mice

Group	0 week	4 week	8 week
Body weight (g)			
Wild ADR	29.1 ± 0.50	29.6 ± 0.41	24.8 ± 0.97^a, c^
Acatalasemic ADR	28.0 ± 0.40	28.2 ± 0.31	24.1 ± 0.84^b, d^
Relative kidney weight (% of body wt)			
Wild ADR	1.68 ± 0.05	1.67 ± 0.04	1.70 ± 0.13
Acatalasemic ADR	1.94 ± 0.04	1.66 ± 0.04	1.30 ± 0.08^b, c, e^
Relative heart weight (% of body wt)			
Wild ADR	0.38 ± 0.02	0.48 ± 0.02	0.48 ± 0.05
Acatalasemic ADR	0.43 ± 0.03	0.52 ± 0.01	0.40 ± 0.03
Blood urea nitrogen (mg/dl)			
Wild ADR	23.8 ± 1.29	26.4 ± 0.77	25.6 ± 4.31
Acatalasemic ADR	26.0 ± 1.02	26.7 ± 2.56	32.6 ± 4.16
Creatinine clearance (μl/min/g BW)			
Wild ADR	2.68 ± 0.81	3.80 ± 0.99	2.10 ± 0.77
Acatalasemic ADR	3.53 ± 1.27	3.21 ± 1.13	1.61 ± 0.61

The values are the means ± SE of 6 to 43 animals in each group

Wild, wild-type mice; Acatalasemic, acatalasemic mice; ADR, adriamycin

^a ^P < 0.05 vs. week 0 in the same group

^b ^P < 0.01 vs. week 0 in the same group

^c ^P < 0.05 vs. week 4 in the same group

^d ^P < 0.01 vs. week 4 in the same group

^e ^P < 0.01 vs. the wild-type ADR group at the same time point

### The increase in albuminuria is significantly accelerated in acatalasemic mice after ADR administration

**Figure 1:**
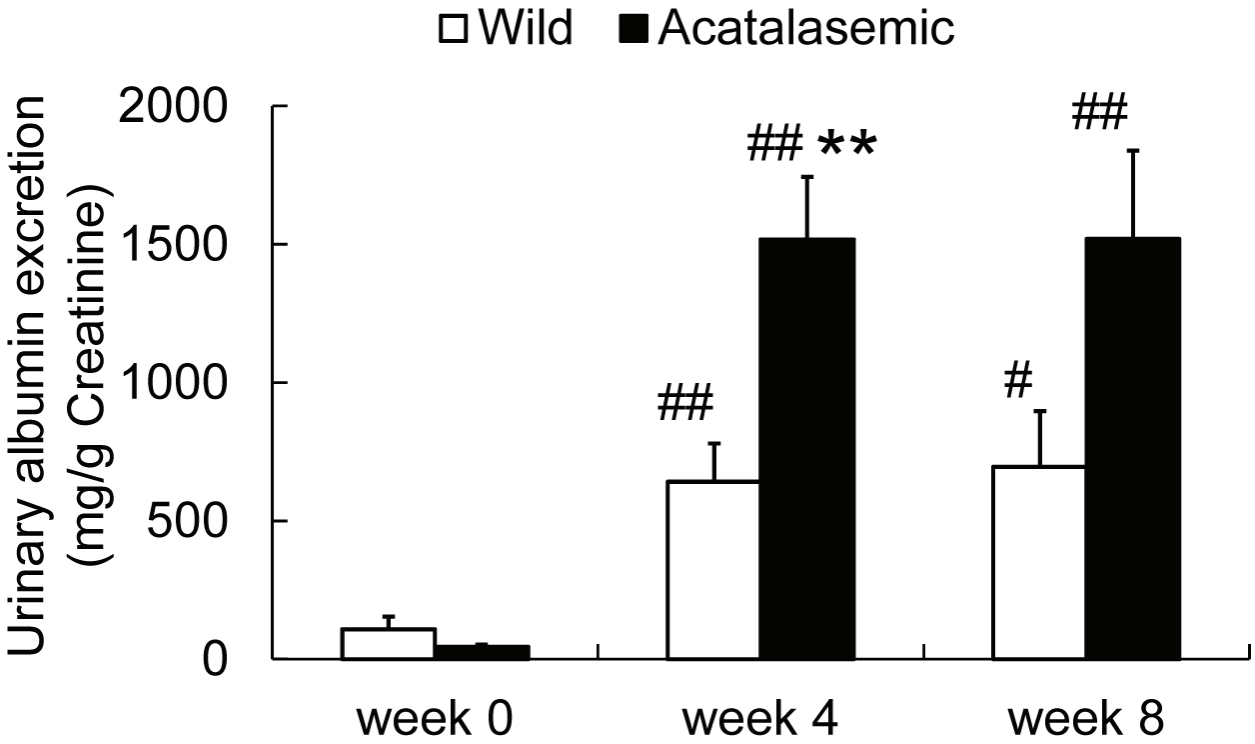
**The changes in urinary albumin excretion (UAE) in wild-type (open bars) and acatalasemic mice (closed bars)**. The UAE significantly increased in both groups at 4 and 8 weeks after Adriamycin (ADR) administration. The elevation of UAE in acatalasemic mice was higher at 4 weeks after ADR administration compared to that in wild-type mice. Each column consists of the means ± SE. N = 9 to 39 animals/group. **: P < 0.01 vs. wild-type ADR mice at the same time point. #: p < 0.05 vs. week 0 in the same group. ##: p < 0.01 vs. week 0 in the same group.

To evaluate the effect of acatalasemia on ADR-induced alterations of biological functions, we measured the parameters of renal function (Table 1) and UAE (Figure 1). The basal levels of BUN and Ccr were not significantly different between the wild-type and acatalasemic mice. In both groups, the renal function did not significantly change during the experimental period. However, UAE was elevated significantly after ADR administration at weeks 4 and 8 in both groups. The UAE in the acatalasemic mice was higher than that in wild-type mice after ADR administration at week 4.

### Acatalasemia accelerates glomerulosclerosis and tubulointerstitial fibrosis

**Figure 2:**
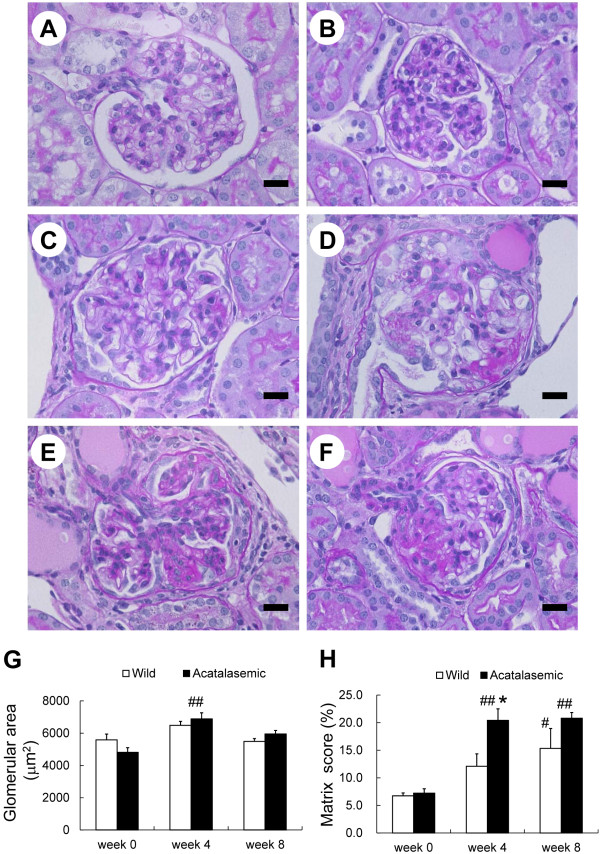
**The renal histology in wild-type and acatalasemic mice**. Light micrographs of a glomerulus of wild-type (*A, C *and *E*) and acatalasemic (*B, D *and *F*) glomeruli at 0, 4 and 8 weeks after adriamycin (ADR) administration are shown. The week 0 glomeruli were microscopically normal. Note that the segmental sclerosis is significant in both acatalasemic and wild-type glomeruli at the later time points. The glomerular area (*G*) and sclerosis index (*H*) of wild-type (open bars) or acatalasemic (closed bars) mice are also shown. *A *through *F*: periodic acid-Schiff stain. Scale bars: 20 μm (*A *through *F*). *G *and *H*: Each column shows the means ± SE. N = 6 to 15 animals/group. *: P < 0.05 vs. wild-type ADR mice at the same time point. #: p < 0.05 vs. week 0 in the same group. ##: p < 0.01 vs. week 0 in the same group.

**Figure 3:**
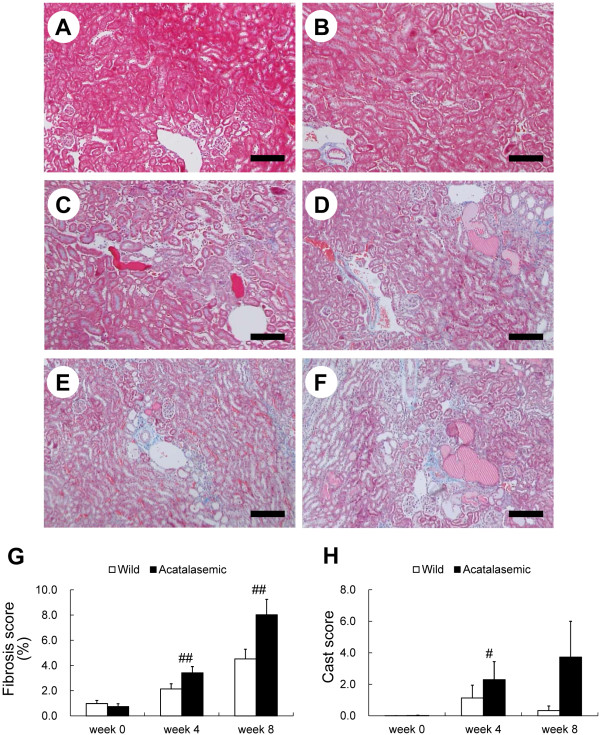
**Light micrographs of the wild-type (*A, C *and *E*) and acatalasemic (*B, D *and *F*) kidney cortex at 0, 4 and 8 weeks after adriamycin (ADR) administration are shown**. Note that interstitial fibrosis significantly developed in the kidneys of the acatalasemic mice. The fibrosis scores (*G*) and cast scores (*H*) of wild-type (open bars) and acatalasemic (closed bars) mice are also shown. *A *through *F*: Masson's trichrome stain. Scale bars: 200 μm (*A *through *F*). *G *and *H*: Each column includes the means ± SE. N = 6 to 15 animals/group. #: p < 0.05 vs. week 0 in the same group. ##: p < 0.01 vs. week 0 in the same group.

At the start of the experiment, no histological abnormalities of the glomeruli were observed at the light microscopic level in either group (Figure 2A and 2B). The glomeruli in the acatalasemic mice were significantly hypertrophied after ADR administration at week 4 (Figure 2D and 2G). In the acatalasemic mice, glomerulosclerosis developed earlier than in wild-type mice, and it was significantly increased at week 4 compared with that in wild-type mice after ADR administration (Figure 2C through *F *and 2*H*). A decrease in the effacement score of the podocyte foot processes was observed in glomeruli of both groups of mice (Additional file 3: Figure S2*A *through *F*), however, there were no significant changes in the effacement scores in either group at week 4 and 8 after ADR administration (Additional file 3: Figure S2*G*). Tubulointerstitial fibrosis developed significantly in the kidneys of acatalasemic mice group (Figure 3B, D, F and 3G) at 4 and 8 weeks after ADR administration, with significant cast formation noted at week 4 (Figure 3H). The tubulointerstitial changes in the kidneys of wild-type mice after ADR administration were not statistically significant compared to that of acatalasemic mice (Figure 3A, C, E, G and 3H).

### Acatalasemia enhances the accumulation of lipid peroxides in the kidneys of the ADR nephropathy model mice

**Figure 4:**
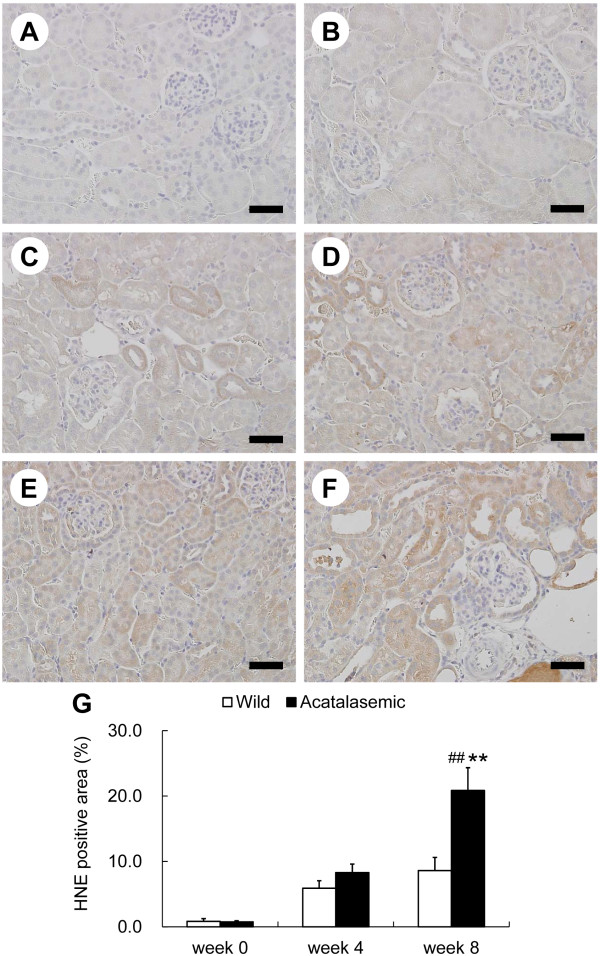
**Immunohistochemical staining of 4-hydroxy-2-nonenal (4-HNE)**. Wild-type (*A, C *and *E*) and acatalasemic (*B, D *and *F*) cortex samples at 0, 4 and 8 weeks after adriamycin (ADR) administration are shown. The 4-HNE positive areas (*G*) of wild-type (open bars) or acatalasemic (closed bars) mice are also shown. Scale bars: 50 μm (*A *through *F*). *G*: Each column shows the means ± SE. N = 6 to 8 animals/group. **: P < 0.01 vs. wild-type ADR mice at the same time point. ##: p < 0.01 vs. week 0 in the same group.

Modification of proteins and lipids by oxidative stress is believed to play a central role in a variety of biological activities, such as apoptosis and extracellular matrix expansion [18,24,25]. The major content of the cell membrane is lipids, and thus, lipid peroxidation may cause renal injury. We next examined whether acatalasemia influenced the lipid peroxidation products in the tubulointerstitial compartment of the kidneys in ADR nephropathy. At the start of the experiment, there was almost no accumulation of 4-HNE in the kidneys of either group (Figure 4A and 4B). There was an increase in the 4-HNE antibody labeling of various patches in the tubules of kidneys after ADR administration in both groups (Figure 4C through 4F). The degree of 4-HNE accumulation in the cortex of the kidneys in acatalasemic mice was significantly higher than that in wild-type mice 8 weeks after ADR administration (Figure 4G).

### The catalase activity in acatalasemic ADR nephropathy kidneys did not significantly change

**Figure 5:**
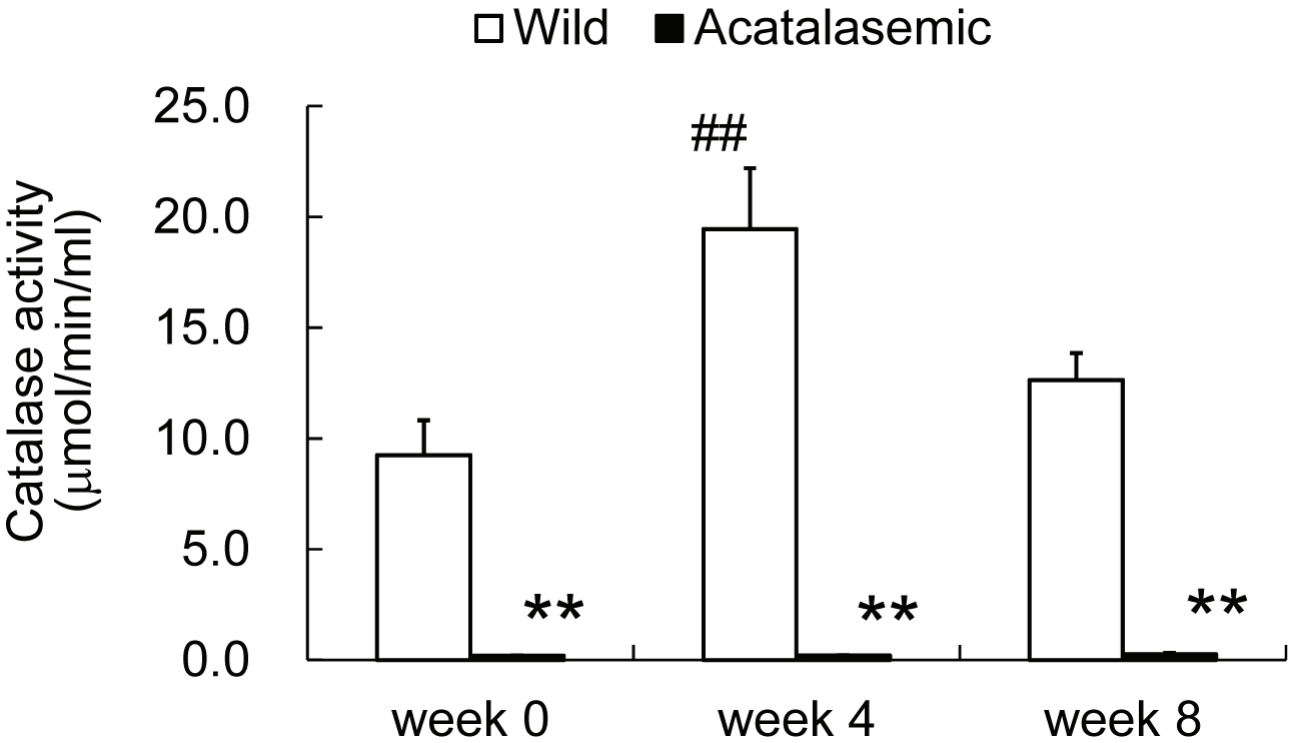
**The renal catalase activities in wild-type (open bars) and acatalasemic mice (closed bars)**. Each column consists of the means ± SE. N = 5 to 10 animals/group. *: P < 0.05 vs. wild-type adriamycin (ADR) mice at the same time point. **: P < 0.01 vs. wild-type ADR mice at the same time point. ##: p < 0.01 vs. week 0 in the same group.

The maintenance of tissue homeostasis requires an appropriate balance between oxidants and antioxidants. Two major antioxidant enzymes, catalase and GPX, are physiologically involved in the detoxification of H_2_O_2_, and thus protect tissues from oxidant-mediated injury. Therefore, we next examined the catalase activity in acatalasemic ADR kidneys. Although the renal catalase activity of the wild-type mice was increased significantly at week 4 after ADR administration, that from the acatalasemic mice was decreased compared with wild-type mice at the start of the experiment, and remained low during the entire experimental period (Figure 5). Since high catalase levels are found in erythrocytes, we compared the catalase activity between saline-perfused and unperfused kidneys after the removal of residual blood. The catalase activity of the perfused kidney was 6.0 ± 0.2% lower than that of the unperfused kidney [17]. Since the catalase activity was enhanced in wild-type mice 4 weeks after ADR administration and was thought to contribute to reactive oxygen elimination, we investigated the levels of other renal antioxidant enzymes, such as GPX and SOD, in wild-type or acatalasemic mice 4 weeks after ADR administration. The activity levels of GPX and SOD did not show any remarkable changes in either of the groups of mice (data not shown). Since SOD catalyzes the conversion of the superoxide anion radical to H_2_O_2 _and O_2_, it has been suggested that the level of production of ROS mediated by SOD may be similar in the ADR-nephropathy kidneys in both groups.

### Toll-like receptor-4 mutation analysis

To examine the background of the mouse strains, we performed a mutation analysis of *Tlr4 *exon 3. *Tlr4 *did not show a C to A transversion due to a missense mutation in the C3H/AnL mouse strain, which was reported to be present in the C3H/HeJ strain (Additional file 4: Figure S3) [23].

## Discussion

In the present study, acatalasemic mouse strains deficient in catalase activity were used as an animal model. These mice were found to be more susceptible to functional and morphological alterations in the kidneys induced by adriamycin than wild-type mice. The level of albuminuria and glomerulosclerosis in the acatalasemic mice after adriamycin injection was significantly higher than that in the wild-type mice. The renal catalase activity in these mice remained low, without compensatory upregulation of GPX or SOD. Collectively, these data suggest that the increased ROS, particularly the hydroxyl radical, resulting from the reduction of catalase activity, may be involved in the acceleration of glomerulosclerosis found under acatalasemic disease conditions.

Although some rat strains show complete susceptibility to ADR, most mouse strains do not. Zheng et al. showed that AKR/J, C3H/HeJ, CBA/J, C57BL/10J, LP/J, SWR/J, SJL/J, and 129S6/SvEvTac mice were resistant to ADR nephropathy, whereas 129S1/SvImJ and BALB/cByJ mice were susceptible [26,27]. They also showed that the susceptible allele for the adriamycin was present in the DOXNPH locus [28]. We did not confirm whether this allele is present in the C3H/AnL mouse strain. Instead, we performed a mutation analysis of the *TLR4 *gene, because it has been thought that TLR4 is involved in progressive renal fibrosis [29]. TLR4 is considered to be the critical component of the LPS receptor complex. In 1998, a point mutation in the *TLR4 *gene was found to be the molecular basis of the LPS hyporesponsiveness in C3H/HeJ mice [23]. The C3H/AnL mice used in the present study did not have this mutation. Therefore, ADR induced mild renal fibrosis in both groups, although the degree of interstitial fibrosis was only significant in the acatalasemic mice.

ADR induced severe albuminuria, thus leading to values ranging from 23 to 226 mg/mgCr in BALB/cj mice and a value of 0.04 mg/gCr in B6/D2 mice [30]. In our study, ADR induced relatively mild albuminuria, with 0.6 mg/mgCr in wild-type and 1.5 mg/mgCr in acatalasemic mice. The C3H/AnL mouse strain is considered to have mild sensitivity to ADR. A single dose of ADR (9.5 mg/kg BW) brought about 40% segmental glomerulosclerosis in the BALB/c mouse strain in a previous study [31]. The wild-type mice which we used in this study had only 15% segmental glomerulosclerosis at week 8 after administration, while the acatalasemic mice had 20% glomerulosclerosis at week 8. This indicates that when the catalase activity is decreased, the resistance to ADR is diminished, and segmental glomerulosclerosis is induced. The number of foot processes of the podocytes did not differ between the mouse groups, indicating that the foot process effacement of the podocytes was affected to a similar degree in both mouse groups. The discrepancy between the level of albuminuria and the degree of foot process effacement may be due to the fact that this evaluation procedure compares only the surviving podocytes in the unsclerosed glomerulus.

Noiri et al. previously investigated the percentage of cortical interstitial volume in the ADR model [19], and Turnberg et al. evaluated tubulointerstitial injury, including cast formation [32]. We evaluated the presence of tubulointerstitial injury in similar analyses, and found significant tubulointerstitial injury only in the acatalasemic mice. The fact that acatalasemia exacerbates pulmonary fibrosis in bleomycin-induced lung injury [33] and chlorhexidine gluconate-induced peritoneal fibrosis [34] was already demonstrated. In this study, we showed that acatalasemia exacerbates renal fibrosis in a murine FSGS model.

The overexpression of catalase prevented albuminuria and interstitial fibrosis in the angiotensinogen transgenic mice [35]. In our study, the deficiency of catalase accelerated the albuminuria and glomerular sclerosis in the ADR nephropathy model. These data suggest that reactive oxygen species may contribute to progressive renal injury. Injac et al. reported that the activity of catalase, which detoxifies hydrogen peroxide to H_2_O, was increased after ADR administration in rats [36]. We measured the catalase activity in the whole kidneys of wild-type mice, and found that its level increased after ADR administration. Therefore, the differences in the albuminuria and the degree of glomerulosclerosis between wild-type and acatalasemic mice may be due to the significant difference of catalase activity in kidneys in these mice and the inability of the acatalasemic mice to increase catalase activity under oxidative stress.

Sheerin et al. reported that ADR-injected mice that died in the complement protein C3+/+ group at 6 weeks had a high serum urea level prior to death [37]. In the current study, all mice survived for at least 4 weeks after the injection of 15 mg/kg BW ADR; however 44.7% of the acatalasemic mice and 40.8% of the wild-type mice died 8 weeks after the administration of ADR. These mortality rates were not significantly different between the two groups (Additional file 1: Figure S1*B*). Since the incidence of albuminuria was analyzed in all of the mice that had survived at 8 weeks, but histological studies were not performed in more than half of the mice that survived at 8 weeks, bias might have been introduced into the analysis. We did not take blood samples or evaluate the serum urea levels in ADR-treated mice that died just before the end of the 8 weeks in this study. In addition, the administration of ADR can cause cardiotoxicity [15] and gastrointestinal toxicity in mice [38], however we could not confirm these toxicities in our experiments.

Acatalasemia is a rare human disease [3,39]. It is unknown whether albuminuria and glomerulosclerosis are related to low catalase activity in humans, although the total antioxidant capacity is correlated with albuminemia, and inversely correlated with proteinuria and anti-DNA antibodies in subjects with lupus nephritis [40]. In addition, apoptosis, which is thought to be related to oxidative stress, correlated with the immunoserological activities of lupus nephritis [41] and idiopathic early FSGS [42]. However, the mechanism(s) by which oxidative stress influences human renal disease is largely unknown. Further studies are needed to elucidate whether and how a low catalase activity in humans influences either albuminuria or glomerulosclerosis associated with kidney diseases.

## Conclusions

We herein demonstrated that ADR induced more albuminuria and glomerulosclerosis in catalase-deficient mice. Treatment with catalase supplementation may contribute to the suppression of progressive renal injury with proteinuria and glomerulosclerosis.

## Competing interests

The authors declare that they have no competing interests.

## Authors' contributions

KT designed the study, carried out the experiments, undertook the data analysis and wrote the manuscript. HS and HM conceived of the study, and participated in its design and coordination, and helped to draft the manuscript. TI, HM, YK and MK participated in experimental sampling. SK and YM undertook the data analyses. NM cooperated with the enzyme activity measurement. DW and KO helped with the animal experiments. All authors read and approved the final manuscript.

## Pre-publication history

The pre-publication history for this paper can be accessed here:

http://www.biomedcentral.com/1471-2369/13/14/prepub

## Supplementary Material

Additional file 1 **Figure S1 The survival rate in the wild-type (open square) or acatalasemic mice (closed square) used for the adriamycin nephropathy model**. (*A*) The mice treated with a dose of 10 mg/kg BW (N = 24, in each group). (B) The mice treated with a dose of 15 mg/kg BW (N = 46 to 66 animals/group). P = 0.56. (*C*) The mice treated with a dose of 20 mg/kg BW (N = 3, in each group). P = 0.43. n.s., not significant. Click here for file

Additional file 2 **Table S1 Primers used for direct sequencing**. Click here for file

Additional file 3 **Figure S2 Electron micrographs of wild-type (*A, C *and *E*) and acatalasemic (*B, D *and *F*) kidneys are shown**. The glomeruli 8 weeks after treatment with the vehicle control showed almost normal foot processes (*A *and *B*). Note the increased podocyte foot process effacement of both kidneys (arrowheads in *C *through *F*) at 4 (C and D) and 8 (*E *and *F*) weeks after adriamycin administration. The effacement score of the podocytes (*G*) of wild-type (open bars) or acatalasemic (closed bars) mice are also shown. Scale bars: 1.0 μm. Each column shows the means ± SE. N = 5 to 6 glomeruli/group. ##: p < 0.01 vs. vehicle control at 8 weeks in the same group. Click here for file

Additional file 4 **Figure S3 The results of the toll-like receptor-4 gene (*Tlr4*) mutation analysis in exon 3 in wild-type (C3H/AnLCsaCsa) and acatalasemic mice (C3H/AnLCsbCsb)**. (A) A diagram of exon 3 along with primer designs for PCR and the sequences. (B) The results of the sequence analysis. *Tlr4 *does not show the missense mutation, a C to A transversion (Pro712His), which was reported in the C3H/HeJ mice strain. Click here for file

## References

[B1] Chance B Sies H Boveris A Hydroperoxide metabolism in mammalian organs Physiol Rev 1979 59 3 527 605 37532 10.1152/physrev.1979.59.3.527

[B2] Zamocky M Furtmuller PG Obinger C Evolution of catalases from bacteria to humans Antioxid Redox Signal 2008 10 9 1527 1548 10.1089/ars.2008.2046 18498226 PMC2959186

[B3] Takahara S Progressive oral gangrene probably due to lack of catalase in the blood (acatalasaemia); report of nine cases Lancet 1952 2 6745 1101 1104 12991731 10.1016/s0140-6736(52)90939-2

[B4] Ogata M Acatalasemia Hum Genet 1991 86 4 331 340 1999334 10.1007/BF00201829

[B5] Ogata M Wang DH Ogino K Mammalian acatalasemia: the perspectives of bioinformatics and genetic toxicology Acta Med Okayama 2008 62 6 345 361 19122680 10.18926/AMO/30951

[B6] Feinstein RN Suter H Jaroslow BN Blood catalsase polymorphism: some immunological aspects Science 1968 159 815 638 640 10.1126/science.159.3815.638 4975476

[B7] Haas M Spargo BH Coventry S Increasing incidence of focal-segmental glomerulosclerosis among adult nephropathies: a 20-year renal biopsy study Am J Kidney Dis 1995 26 5 740 750 10.1016/0272-6386(95)90437-9 7485126

[B8] Lavin PJ Gbadegesin R Damodaran TV Winn MP Therapeutic targets in focal and segmental glomerulosclerosis Curr Opin Nephrol Hypertens 2008 17 4 386 392 10.1097/MNH.0b013e32830464f4 18660675 PMC2674376

[B9] Chen A Wei CH Sheu LF Ding SL Lee WH Induction of proteinuria by adriamycin or bovine serum albumin in the mouse Nephron 1995 69 3 293 300 10.1159/000188473 7753263

[B10] Chen A Sheu LF Ho YS Lin YF Chou WY Chou TC Lee WH Experimental focal segmental glomerulosclerosis in mice Nephron 1998 78 4 440 452 10.1159/000044974 9578071

[B11] Deman A Ceyssens B Pauwels M Zhang J Houte KV Verbeelen D Van den Branden C Altered antioxidant defence in a mouse adriamycin model of glomerulosclerosis Nephrol Dial Transplant 2001 16 1 147 150 10.1093/ndt/16.1.147 11209009

[B12] Oteki T Nagase S Shimohata H Hirayama A Ueda A Yokoyama H Yoshimura T Nitric oxide protection against adriamycin-induced tubulointerstitial injury Free Radic Res 2008 42 2 154 161 10.1080/10715760701840047 18297608

[B13] Guo J Ananthakrishnan R Qu W Lu Y Reiniger N Zeng S Ma W Rosario R Yan SF Ramasamy R RAGE mediates podocyte injury in adriamycin-induced glomerulosclerosis J Am Soc Nephrol 2008 19 5 961 972 10.1681/ASN.2007101109 18256352 PMC2386730

[B14] Koshikawa M Mukoyama M Mori K Suganami T Sawai K Yoshioka T Nagae T Yokoi H Kawachi H Shimizu F Role of p38 mitogen-activated protein kinase activation in podocyte injury and proteinuria in experimental nephrotic syndrome J Am Soc Nephrol 2005 16 9 2690 2701 10.1681/ASN.2004121084 15987752

[B15] Shioji K Kishimoto C Nakamura H Masutani H Yuan Z Oka S Yodoi J Overexpression of thioredoxin-1 in transgenic mice attenuates adriamycin-induced cardiotoxicity Circulation 2002 106 11 1403 1409 10.1161/01.CIR.0000027817.55925.B4 12221060

[B16] Kobayashi M Sugiyama H Wang DH Toda N Maeshima Y Yamasaki Y Masuoka N Yamada M Kira S Makino H Catalase deficiency renders remnant kidneys more susceptible to oxidant tissue injury and renal fibrosis in mice Kidney Int 2005 68 3 1018 1031 10.1111/j.1523-1755.2005.00494.x 16105032

[B17] Sunami R Sugiyama H Wang DH Kobayashi M Maeshima Y Yamasaki Y Masuoka N Ogawa N Kira S Makino H Acatalasemia sensitizes renal tubular epithelial cells to apoptosis and exacerbates renal fibrosis after unilateral ureteral obstruction Am J Physiol Renal Physiol 2004 286 6 F1030 F1038 10.1152/ajprenal.00266.2003 14722014

[B18] Kikumoto Y Sugiyama H Inoue T Morinaga H Takiue K Kitagawa M Fukuoka N Saeki M Maeshima Y Wang DH Sensitization to alloxan-induced diabetes and pancreatic cell apoptosis in acatalasemic mice Biochim Biophys Acta 2010 1802 2 240 246 19883754 10.1016/j.bbadis.2009.10.009

[B19] Noiri E Nagano N Negishi K Doi K Miyata S Abe M Tanaka T Okamoto K Hanafusa N Kondo Y Efficacy of darbepoetin in doxorubicin-induced cardiorenal injury in rats Nephron Exp Nephrol 2006 104 1 e6 e14 10.1159/000093258 16707910

[B20] Ikeda Y Tanaka H Esaki M Effects of gestational diethylstilbestrol treatment on male and female gonads during early embryonic development Endocrinology 2008 149 8 3970 3979 10.1210/en.2007-1599 18436715 PMC2488225

[B21] Uchida HA Sugiyama H Takiue K Kikumoto Y Inoue T Makino H Development of Angiotensin II-induced Abdominal Aortic Aneurysms Is Independent of Catalase in Mice J Cardiovasc Pharmacol 2011 58 6 633 638 10.1097/FJC.0b013e3182317196 21885993

[B22] Noguchi N Rimbara E Kato A Tanaka A Tokunaga K Kawai T Takahashi S Sasatsu M Detection of mixed clarithromycin-resistant and -susceptible Helicobacter pylori using nested PCR and direct sequencing of DNA extracted from faeces J Med Microbiol 2007 56 Pt 9 1174 1180 17761479 10.1099/jmm.0.47302-0

[B23] Poltorak A He X Smirnova I Liu MY Van Huffel C Du X Birdwell D Alejos E Silva M Galanos C Defective LPS signaling in C3H/HeJ and C57BL/10ScCr mice: mutations in Tlr4 gene Science 1998 282 5396 2085 2088 9851930 10.1126/science.282.5396.2085

[B24] Makino H Sugiyama H Kashihara N Apoptosis and extracellular matrix-cell interactions in kidney disease Kidney Int Suppl 2000 77 S67 S75 10997693

[B25] Sugiyama H Kashihara N Maeshima Y Okamoto K Kanao K Sekikawa T Makino H Regulation of survival and death of mesangial cells by extracellular matrix Kidney Int 1998 54 4 1188 1196 10.1046/j.1523-1755.1998.00116.x 9767534

[B26] Zheng Z Pavlidis P Chua S D'Agati VD Gharavi AG An ancestral haplotype defines susceptibility to doxorubicin nephropathy in the laboratory mouse J Am Soc Nephrol 2006 17 7 1796 1800 10.1681/ASN.2005121373 16775033

[B27] Pippin JW Brinkkoetter PT Cormack-Aboud FC Durvasula RV Hauser PV Kowalewska J Krofft RD Logar CM Marshall CB Ohse T Inducible rodent models of acquired podocyte diseases Am J Physiol Renal Physiol 2009 296 2 F213 F229 18784259 10.1152/ajprenal.90421.2008

[B28] Zheng Z Schmidt-Ott KM Chua S Foster KA Frankel RZ Pavlidis P Barasch J D'Agati VD Gharavi AG A Mendelian locus on chromosome 16 determines susceptibility to doxorubicin nephropathy in the mouse Proc Natl Acad Sci USA 2005 102 7 2502 2507 10.1073/pnas.0409786102 15699352 PMC549022

[B29] Anders HJ Banas B Schlondorff D Signaling danger: toll-like receptors and their potential roles in kidney disease J Am Soc Nephrol 2004 15 4 854 867 10.1097/01.ASN.0000121781.89599.16 15034087

[B30] Brideau G Doucet A Over-expression of adenosine deaminase in mouse podocytes does not reverse puromycin aminonucleoside resistance BMC Nephrol 2010 11 15 10.1186/1471-2369-11-15 20649959 PMC2915970

[B31] Wu H Wang YM Wang Y Hu M Zhang GY Knight JF Harris DC Alexander SI Depletion of gammadelta T cells exacerbates murine adriamycin nephropathy J Am Soc Nephrol 2007 18 4 1180 1189 10.1681/ASN.2006060622 17329577

[B32] Turnberg D Lewis M Moss J Xu Y Botto M Cook HT Complement activation contributes to both glomerular and tubulointerstitial damage in adriamycin nephropathy in mice J Immunol 2006 177 6 4094 4102 16951374 10.4049/jimmunol.177.6.4094

[B33] Odajima N Betsuyaku T Nagai K Moriyama C Wang DH Takigawa T Ogino K Nishimura M The role of catalase in pulmonary fibrosis Respir Res 2010 11 183 10.1186/1465-9921-11-183 21190578 PMC3022724

[B34] Fukuoka N Sugiyama H Inoue T Kikumoto Y Takiue K Morinaga H Nakao K Maeshima Y Asanuma M Wang DH Increased susceptibility to oxidant-mediated tissue injury and peritoneal fibrosis in acatalasemic mice Am J Nephrol 2008 28 4 661 668 10.1159/000121357 18337633

[B35] Godin N Liu F Lau GJ Brezniceanu ML Chenier I Filep JG Ingelfinger JR Zhang SL Chan JS Catalase overexpression prevents hypertension and tubular apoptosis in angiotensinogen transgenic mice Kidney Int 2010 77 12 1086 1097 10.1038/ki.2010.63 20237455

[B36] Injac R Boskovic M Perse M Koprivec-Furlan E Cerar A Djordjevic A Strukelj B Acute doxorubicin nephrotoxicity in rats with malignant neoplasm can be successfully treated with fullerenol C60(OH)24 via suppression of oxidative stress Pharmacol Rep 2008 60 5 742 749 19066422

[B37] Sheerin NS Risley P Abe K Tang Z Wong W Lin T Sacks SH Synthesis of complement protein C3 in the kidney is an important mediator of local tissue injury FASEB J 2008 22 4 1065 1072 18039928 10.1096/fj.07-8719com

[B38] Morelli D Menard S Colnaghi MI Balsari A Oral administration of anti-doxorubicin monoclonal antibody prevents chemotherapy-induced gastrointestinal toxicity in mice Cancer Res 1996 56 9 2082 2085 8616854

[B39] Goth L Eaton JW Hereditary catalase deficiencies and increased risk of diabetes Lancet 2000 356 9244 1820 1821 10.1016/S0140-6736(00)03238-4 11117918

[B40] Moroni G Novembrino C Quaglini S De Giuseppe R Gallelli B Uva V Montanari V Messa P Bamonti F Oxidative stress and homocysteine metabolism in patients with lupus nephritis Lupus 2010 19 1 65 72 10.1177/0961203309346906 19933721

[B41] Makino H Sugiyama H Yamasaki Y Maeshima Y Wada J Kashihara N Glomerular cell apoptosis in human lupus nephritis Virchows Arch 2003 443 1 67 77 10.1007/s00428-003-0827-x 12750884

[B42] Erkan E Garcia CD Patterson LT Mishra J Mitsnefes MM Kaskel FJ Devarajan P Induction of renal tubular cell apoptosis in focal segmental glomerulosclerosis: roles of proteinuria and Fas-dependent pathways J Am Soc Nephrol 2005 16 2 398 407 10.1681/ASN.2003100861 15601749

