# Supplementary figures and images for: Acatalasemic mice are mildly susceptible to adriamycin nephropathy and exhibit increased albuminuria and glomerulosclerosis
Source: BMC Nephrol. 2012 Mar 25;13:14. doi: 10.1186/1471-2369-13-14 (PMC3329410; doi:10.1186/1471-2369-13-14)

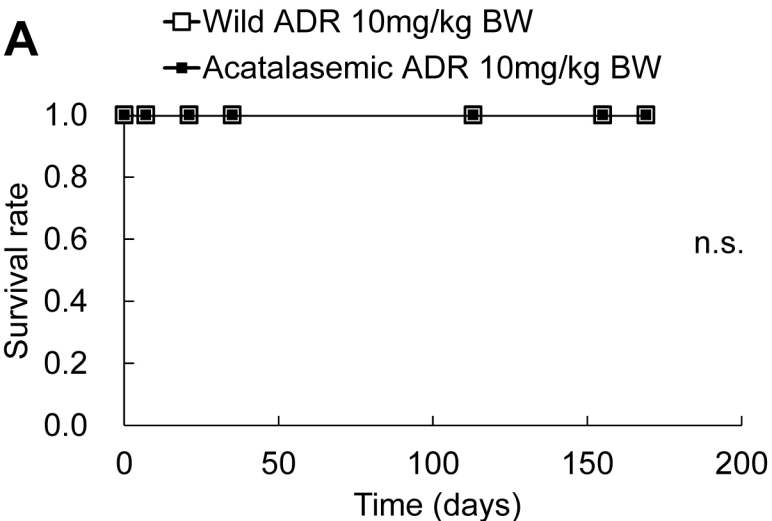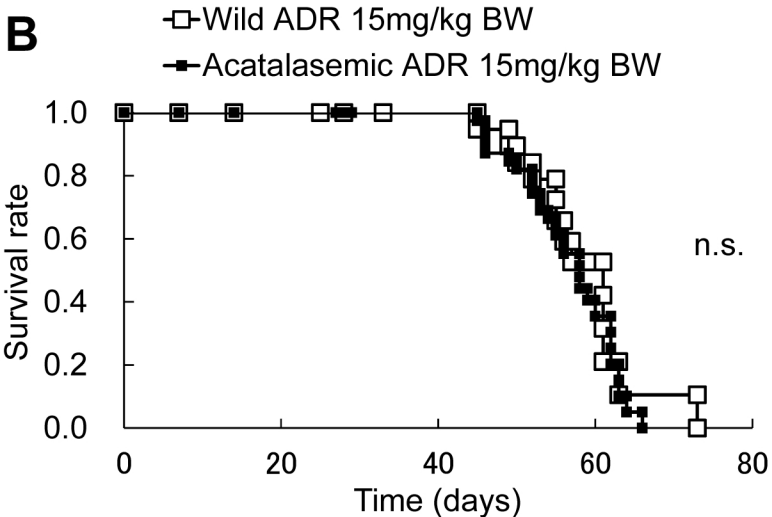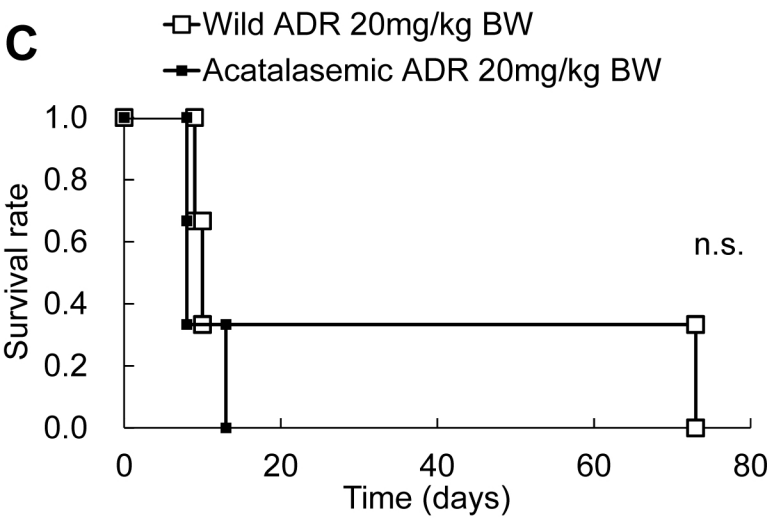

Supplement: Additional file 1 — Figure S1 The survival rate in the wild-type (open square) or acatalasemic mice (closed square) used for the adriamycin nephropathy model. (A) The mice treated with a dose of 10 mg/kg BW (N = 24, in each group). (B) The mice treated with a dose of 15 mg/kg BW (N = 46 to 66 animals/group). P = 0.56. (C) The mice treated with a dose of 20 mg/kg BW (N = 3, in each group). P = 0.43. n.s., not significant. [file 1471-2369-13-14-S1.PDF]

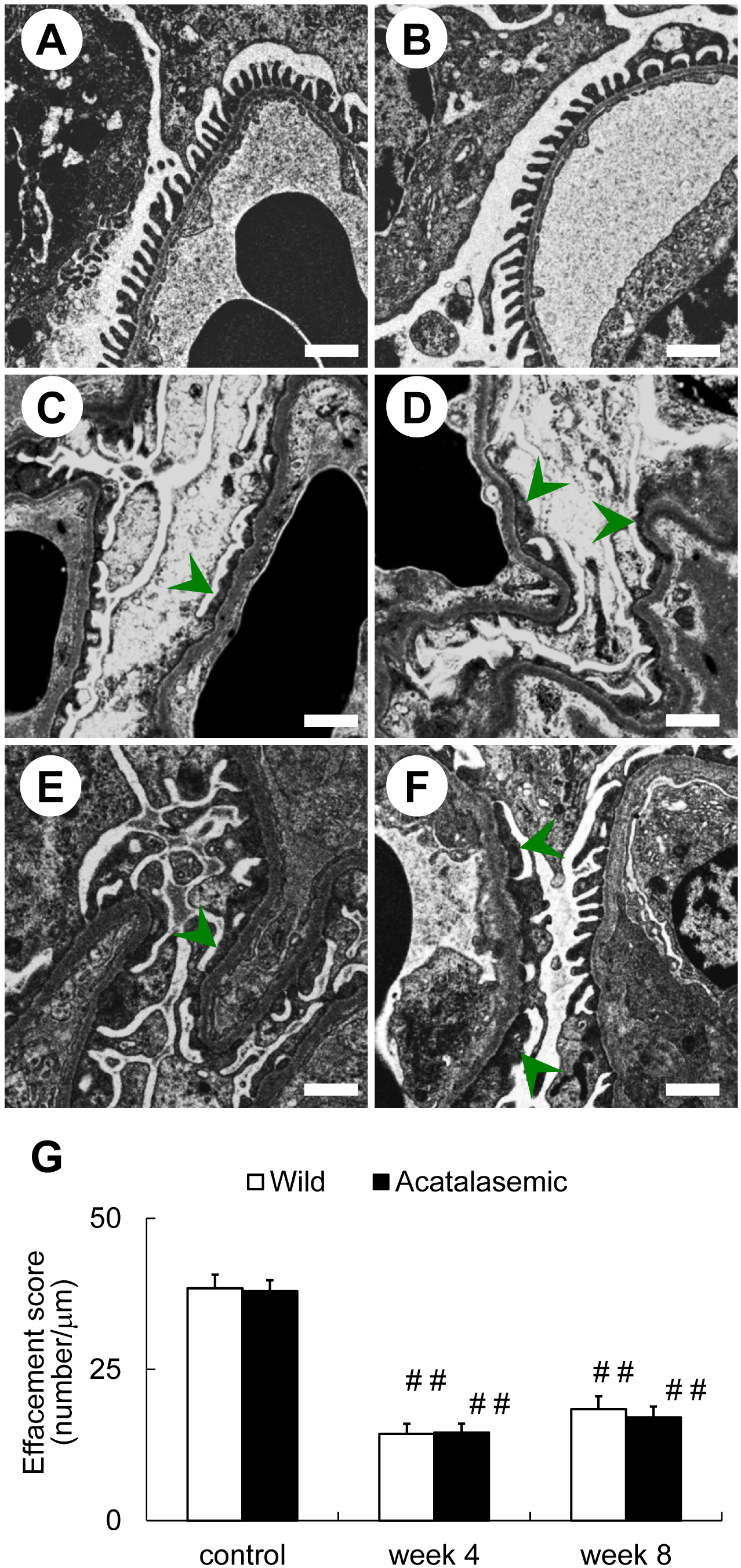

Supplement: Additional file 3 — Figure S2 Electron micrographs of wild-type (A, C and E) and acatalasemic (B, D and F) kidneys are shown. The glomeruli 8 weeks after treatment with the vehicle control showed almost normal foot processes (A and B). Note the increased podocyte foot process effacement of both kidneys (arrowheads in C through F) at 4 (C and D) and 8 (E and F) weeks after adriamycin administration. The effacement score of the podocytes (G) of wild-type (open bars) or acatalasemic (closed bars) mice are also shown. Scale bars: 1.0 μm. Each column shows the means ± SE. N = 5 to 6 glomeruli/group. ##: p < 0.01 vs. vehicle control at 8 weeks in the same group. [file 1471-2369-13-14-S3.JPEG]
