# Supplementary material for: Acatalasemic mice are mildly susceptible to adriamycin nephropathy and exhibit increased albuminuria and glomerulosclerosis
Source: BMC Nephrol. 2012 Mar 25;13:14. doi: 10.1186/1471-2369-13-14 (PMC3329410; doi:10.1186/1471-2369-13-14)
Supplement: Additional file 2 — Table S1 Primers used for direct sequencing. [file 1471-2369-13-14-S2.DOC]

**SUPPLEMENTARY INFORMATION**

**Acatalasemic Mice Are Susceptible to Adriamycin Nephropathy and Exhibit Increased Albuminuria and Glomerulosclerosis**

**Keiichi Takiue^1^, M.D. Hitoshi Sugiyama^2^, M.D. Ph.D. Tatsuyuki Inoue^1, 3^, M.D. Ph.D. Hiroshi Morinaga^2^, M.D. Yoko Kikumoto^1^ ,M.D. Ph.D. Masashi Kitagawa^1^, M.D. Shinji Kitamura^1^, M.D. Ph.D. Yohei Maeshima^1^, M.D. Ph.D. Da-Hong Wang^4^, M.D. Ph.D. Noriyoshi Masuoka^5^, M.D. Ph.D. Keiki Ogino^4^, M.D. Ph.D. Hirofumi Makino^1^, M.D. Ph.D.**

^1^Department of Medicine and Clinical Science, ^2^Center for CKD and Peritoneal Dialysis, ^4^Public Health, Okayama University Graduate School of Medicine, Dentistry and Pharmaceutical Sciences, 2-5-1 Shikata-cho, Kita-ku, Okayama 700-8558, Japan

**^3^**Center for iPS **C**ell Research and Application, Kyoto University, Kyoto, Japan

^5^Department of Life Science, Okayama University of Science, 1-1, Ridai-cho, Kita-ku, Okayama 700-0005, Japan

**Contents:**

**Supplementary Figure Legends S1-S3**

**Supplementary Tables S1**

**Supplementary Figure Legends**

**Supplementary Figure S1**: The survival rate in the wild-type (open square) or acatalasemic mice (closed square) used for the adriamycin nephropathy model. (*A*) The mice treated with a dose of 10 mg/kg BW (N =24, in each group). (B) The mice treated with a dose of 15 mg/kg BW (N = 46 to 66 animals/group). P = 0.56. (*C*) The mice treated with a dose of 20 mg/kg BW (N = 3, in each group). P = 0.43. n.s., not significant.

**Supplementary Figure S2**: Electron micrographs of wild-type (*A*, *C* and *E*) and acatalasemic (*B*, *D* and *F*) kidneys are shown. The glomeruli 8 weeks after treatment with the vehicle control showed almost normal foot processes (*A* and *B*). Note the increased podocyte foot process effacement of both kidneys (arrowheads in *C* through *F*) at 4 (C and D) and 8 (*E* and *F*) weeks after adriamycin administration. The effacement score of the podocytes (*G*) of wild-type (open bars) or acatalasemic (closed bars) mice are also shown. Scale bars: 1.0 μm. Each column shows the means ± SE. N = 5 to 6 glomeruli/group. ##: p<0.01 vs. vehicle control at 8 weeks in the same group.

**Supplementary Figure S3**: The results of the toll-like receptor-4 gene (*Tlr4*) mutation analysis in exon 3 in wild-type (C3H/AnLCs^a^Cs^a^) and acatalasemic mice (C3H/AnLCs^b^Cs^b^). (*A*) A diagram of exon 3 along with primer designs for PCR and the sequences. (*B*) The results of the sequence analysis. *Tlr4* does not show the missense mutation, a C to A transversion (Pro712His), which was reported in the C3H/HeJ mice strain.

Supplementary Table S1. Primers used for direct sequencing

| Primer | Tm (℃) | Sequence (5’→3’) |
| --- | --- | --- |
| First-round |  |  |
| TLR4-ex3-nest-F | 59.47 | AGATGAATACCTCCTTAGTGTTGGA |
| TLR4-ex3-nest-R | 61.18 | ATTCCAGGTAGGTGTTTCTGCTAAG |
| Second-round |  |  |
| TLR4-ex3-F | 60.52 | ATTGTGGTATCCACTGTAGCATTTC |
| TLR4-ex3-R | 60.87 | ACTTCTCAACCTTCTCAAGGACAAT |

GenBank accession no. NM_021297. TLR, toll-like receptor.
