# Supplementary material for: Acatalasemic mice are mildly susceptible to adriamycin nephropathy and exhibit increased albuminuria and glomerulosclerosis
Source: BMC Nephrol. 2012 Mar 25;13:14. doi: 10.1186/1471-2369-13-14 (PMC3329410; doi:10.1186/1471-2369-13-14)
Supplement: Additional file 4 — Figure S3 The results of the toll-like receptor-4 gene (Tlr4) mutation analysis in exon 3 in wild-type (C3H/AnLCsaCsa) and acatalasemic mice (C3H/AnLCsbCsb). (A) A diagram of exon 3 along with primer designs for PCR and the sequences. (B) The results of the sequence analysis. Tlr4 does not show the missense mutation, a C to A transversion (Pro712His), which was reported in the C3H/HeJ mice strain. [file 1471-2369-13-14-S4.PDF]

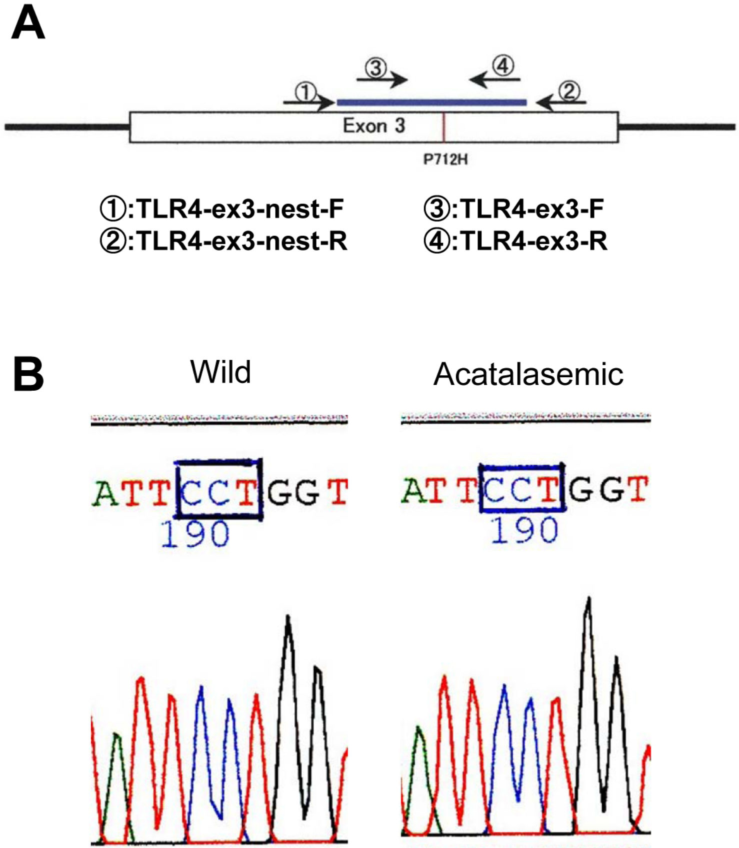
A
③
④
①
②
Exon 3
P712H
①:TLR4-ex3-nest-F
②:TLR4-ex3-nest-R
③:TLR4-ex3-F
④:TLR4-ex3-R
B
Wild
Acatalasemic
ATTCCTGGT
190
ATTCCTGGT
190
